# Factors Associated with Nursing Home Placement of All Patients Admitted for Inpatient Rehabilitation in Singapore Community Hospitals from 1996 to 2005: A Disease Stratified Analysis

**DOI:** 10.1371/journal.pone.0082697

**Published:** 2013-12-23

**Authors:** Cynthia Chen, Nasheen Naidoo, Benjamin Er, Angela Cheong, Ngan Phoon Fong, Choo Yian Tay, Kin Ming Chan, Boon Yeow Tan, Edward Menon, Chye Hua Ee, Kok Keng Lee, Yee Sien Ng, Yik Ying Teo, Gerald C. H. Koh

**Affiliations:** 1 Saw Swee Hock School of Public Health, National University of Singapore, National University Health System, Singapore, Singapore; 2 Planning Division, Agency for Integrated Care, Singapore, Singapore; 3 Medical Services, Ang Mo Kio Thye Hua Kwan Hospital, Singapore, Singapore; 4 Medical Services, St Luke’s Hospital, Singapore, Singapore; 5 Medical Services, St Andrew’s Community Hospital, Singapore, Singapore; 6 Bright Vision Hospital, Singapore, Singapore; 7 Department of Rehabilitation Medicine, Singapore General Hospital, Singapore, Singapore; 8 Genome Institute of Singapore, Agency for Science, Technology, and Research, Singapore, Singapore; 9 Graduate School for Integrative Science and Engineering, National University of Singapore, Singapore, Singapore; 10 Department of Statistics and Applied Probability, National University of Singapore, Singapore, Singapore; University of Glasgow, United Kingdom

## Abstract

**Objectives:**

To (1) identify social and rehabilitation predictors of nursing home placement, (2) investigate the association between effectiveness and efficiency in rehabilitation and nursing home placement of patients admitted for inpatient rehabilitation from 1996 to 2005 by disease in Singapore.

**Design:**

National data were retrospectively extracted from medical records of community hospital.

**Data Sources:**

There were 12,506 first admissions for rehabilitation in four community hospitals. Of which, 8,594 (90.3%) patients were discharged home and 924 (9.7%) patients were discharged to a nursing home. Other discharge destinations such as sheltered home (n = 37), other community hospital (n = 31), death in community hospital (n = 12), acute hospital (n = 1,182) and discharge against doctor’s advice (n = 24) were excluded.

**Outcome Measure:**

Nursing home placement.

**Results:**

Those who were discharged to nursing home had 33% lower median rehabilitation effectiveness and 29% lower median rehabilitation efficiency compared to those who were discharged to nursing homes. Patients discharged to nursing homes were significantly older (mean age: 77 vs. 73 years), had lower mean Bathel Index scores (40 vs. 48), a longer median length of stay (40 vs. 33 days) and a longer time to rehabilitation (19 vs. 15 days), had a higher proportion without a caregiver (28 vs. 7%), being single (21 vs. 7%) and had dementia (23 vs. 10%). Patients admitted for lower limb amputation or falls had an increased odds of being discharged to a nursing home by 175% (p<0.001) and 65% (p = 0.043) respectively compared to stroke patients.

**Conclusions:**

In our study, the odds of nursing home placement was found to be increased in Chinese, males, single or widowed or separated/divorced, patients in high subsidy wards for hospital care, patients with dementia, without caregivers, lower functional scores at admission, lower rehabilitation effectiveness or efficiency at discharge and primary diagnosis groups such as fractures, lower limb amputation and falls in comparison to strokes.

## Introduction

There is an increasing global demand for nursing home beds due to the growing ageing population. [1] The need for nursing home institutionalization is often complex and driven by many factors such as the patient’s age, medical conditions, socio-demographic variables, cost issues and caregiver availability. However, these factors may vary in importance between different diseases.

Singapore is a rapidly ageing society. With an increasing life expectancy at birth of 84.3 years for women and 79.6 years for men in 2012, [2] the number of elderly aged 65 years and above will triple to over 900,000 by 2030. [3] There are altogether 8 public hospitals in Singapore which comprise of 6 acute hospitals, a women’s and children’s hospital and a psychiatric hospital. The general hospitals provide multi-disciplinary acute inpatient and specialist outpatient services and a 24-hour emergency department. [4] Community hospitals in Singapore were introduced as part of the intermediate and long term care for the convalescent sick and aged who do not require the care of the acute hospitals. Although community hospitals provide mainly rehabilitation, they also offer sub-acute, chronic sick and respite care. Community hospitals are distinct from acute hospitals as they do not offer acute emergency services or provide expensive ancillary services such as computerized tomography or magnetic resonance imaging services. According to MOH guidelines, it is generally recommended that rehabilitation units in acute hospitals cater to younger patients where the goal is to return the patient to the workforce while rehabilitation in community hospitals cater to older patients where the goal is to return the patient to their homes. [5] As a result, staff in rehabilitation units in acute hospitals are trained in specialized fields such as traumatic spinal injury while staff in community hospitals are trained in geriatric medicine.

In general, patients are directly admitted to these community hospitals from acute hospitals and receive inpatient rehabilitation during their stay. Most patients are discharged to their own homes but a few are transferred to a nursing home. A minority of patients are transferred back to the acute hospital, usually within the first week of admission, because their medical status deteriorates beyond the community hospital’s capability to manage them safely. Patients transferred to community hospitals are usually newly disabled elderly who suffered an acute medical condition requiring rehabilitation. The common principal diagnoses for admission include stroke, hip fractures, de-conditioning from medical illness or surgery and amputations [6].

Nursing homes are run by either the private sector or the voluntary welfare sector (VWOs) in Singapore. Elderly persons may be admitted into a nursing home if they require daily skilled nursing care or assistance in activities of daily living (ADL) or have no caregiver to look after them at home. [7] To qualify for nursing home care they must be semi-ambulant, wheel-chair or bed bound. Those with medical conditions (e.g. stroke, diabetes mellitus with complications, head or spinal injury) are also eligible for nursing home care. Nursing homes provide a range of services to meet the needs of their residents, including medical care, nursing care, physiotherapy, dietary services and dental care. Some nursing homes provide care for persons with special needs such as dementia and persons with stable psychiatric conditions. Respite care is also available at some nursing homes where provision for short-term care of a few weeks can be arranged. It is projected that by 2030, the number of elderly who are semi-ambulant or non-ambulant will double to 117,000, while dementia cases would almost triple to 80,000. [8] Thus there is a need in Singapore to ensure a continuum of care from an acute to the community setting to serve the increasing ageing population, especially those with chronic diseases.

Several studies have consistently reported that being female, marital status (lack of a spouse), advanced age, minority race, and poverty status are determinants of nursing home admission.[9]–[12] Risk of nursing home placement is increased with poorer socioeconomic status. [9], [13] In addition, social support and caregiver support are associated with nursing home placement [14], [15].

Most major studies to date have indicated that impairment in activities of daily living (ADL) significantly increases the risk of nursing home placement.[9], Health-related factors such as functional disabilities were found to be more important predictors than demographic profile or support system. [20] Shapiro at el. reported that without adding an ADL problem, the chances of institutionalisation for an older patient remained below 50%, even when all other risk factors (i.e. aged ≥85, no spouse living together, recent hospital admission, living in retirement housing, mental impairment) were present. [11] A local study showed similar findings where 43% of residents admitted to the nursing home were due to both medical and social factors, with malnutrition, urinary incontinence, falls, functional decline, and impaired vision or hearing identified as common variables. [21] Few studies have documented the association between rehabilitation discharge outcomes such as rehabilitation effectiveness and efficiency, and nursing home placement compared across different diseases. A recent study by Koh et al [22] observed trade-offs between rehabilitation effectiveness and efficiency with respect to hospital admission Barthel Index score and length of stay for stroke patients. As length of stay increased, patients performed better in rehabilitation effectiveness at the expense of rehabilitation efficiency. It will be useful to determine whether these rehabilitation outcomes predict nursing home placements across different diseases and the socio-demographic characteristics of patients admitted for inpatient rehabilitation in Singapore community hospitals and nursing home placement, in order to better prepare patients and their caregivers for post-acute care, inform public policy, and improve program planning.

## Methods

### Data Extraction

Data from medical records were retrospectively extracted for all patients admitted into the four community hospitals across Singapore for rehabilitation from 2 January 1996 to 31 December 2005. Hospital A is a 200-bedded hospital which opened in 1993. Hospital B is a 185-bedded community hospital which opened in 1996. Hospital C is the oldest community hospital in Singapore – it opened in 1992 and only had 40 beds till 2005 when it moved to a new premise and expanded its bed capacity to 200 beds. Hospital D opened in 2003 and currently has 120 beds.

Community hospitals in Singapore provide inpatient rehabilitation for the needs of Singaporeans. [23] As per Singapore’s Ministry of Health guidelines, community hospitals ensure that these patients achieve their optimal health potential before discharge. [24] Rehabilitation is provided each weekday for approximately one hour. This includes individualized physical, occupational and speech therapy as appropriate. Data extraction from non-computerized medical records was manually performed from November 2005 to August 2008 by four research nurses who were trained and supervised by the last author (GK). Multiple iterations of data cleaning and verification were performed. A 10% random sample of patients was subsequently analyzed for data extraction accuracy by an independent physician and the error rate was 0.07%. The study was approved by the National University of Singapore Institutional Review Board (NUS-IRB) and ethics committees of Ang Mo Kio Thye Hua Kwan Hospital, Bright Vision Hospital, St Andrew’s Community Hospital and St Luke’s Hospital. Written informed consent of the patient was waived by approving NUS-IRB. The corresponding author and all research nurses have taken the oath of confidentiality under Singapore’s Official Secrets Act and only the minimum number of research personnel had access to the de-identified dataset.

### Data Management

For our study, we only included first admissions for rehabilitation. Independent variables were socio-demographic variables and variables related to caregiver factors. In Singapore, only patients staying in C class (non air-conditioned 8-bedded) or B2 class (non air-conditioned 6-bedded) wards receive government subsidies for hospital stay (75% and 50% subsidy respectively); patients in higher class (i.e. air-conditioned four bedded to single bedded) wards do not receive subsidies. We dichotomized government subsidy levels into C class versus B2 class and above, as C class patients best represent the lower socio-economic group in our population.

### Outcome Measures

Length of stay was calculated as the total number of days from hospital admission to discharge. Functional status was assessed using the Shah-modified Barthel Index (BI) by all rehabilitation hospitals in Singapore as recommended by our local Ministry of Health. [24] The Shah-modified BI has a range from zero to 100, with five sub-categories for each of the 10 activities of daily living category, and 100 possible discrete values [25] where higher scores reflect greater independence in function. The five subcategories are (1) patients who were unable to perform the task, (2) patients greatly dependent or unsafe to perform the task without caregiver’s presence, (3) patients requiring moderate assistance to complete the task, (4) patients requiring minimal assistance and (4) patients who are fully independent. As per the Singapore Ministry of Health’s requirements, admission BI should be scored within 48 hours of admission and at least every two weeks until discharge. [24] These were assessed by both physiotherapists and occupational therapists. The first BI score recorded was taken as the admission BI and last BI score was recorded as the discharge BI. Absolute functional gain (Absolute-FG) is the amount of improvement achieved with rehabilitation calculated as:
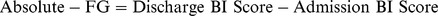



Rehabilitation outcomes can be measured in terms of rehabilitation effectiveness (R-effectiveness) [26] and rehabilitation efficiency (R-efficiency) [26], [27]. Patients with negative R-effectiveness and R-efficiency measures have declined in functional status.

Rehabilitation effectiveness was a concept first suggested by Heinemann et al in 1987 who reported the mean percentage of achieved rehabilitation potential of their study population as 55% (standard deviation, SD = 15%). [28] However, it was Shah et al who coined the term Rehabilitation effectiveness later in 1990. [29] Expressed as a percentage reflecting the proportion of potential improvement actually achieved during rehabilitation, it can be calculated using the formula:
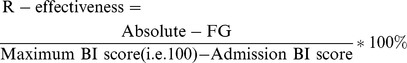



The concept of rehabilitation efficiency was also first suggested by Heinemann et al in 1987 who reported the mean rehabilitation efficiency index of their study population as 0.6 units per day (SD = 0.5 units per day) using the BI. [28] Later, Shah et al renamed this concept to simply Rehabilitation efficiency. [29] It is the amount of functional improvement divided by the duration of rehabilitation. It can be regarded as the average increase in the score of a functional assessment tool per 30 days and is calculated using the following formula:
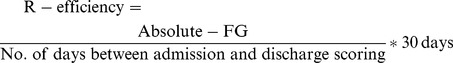



The discharge destination of patients was collected from patient records at community hospitals and coded as home, acute hospital, nursing home, sheltered home, discharge against doctor’s advice, death and others. Sheltered homes in Singapore are residential facilities which cater to the needs of ambulant senior citizens and provide some support services to maintain their independence within the community. We only selected patients who were discharged home or to a nursing home, and excluded other discharge destinations.

All analyses were adjusted for the primary diagnosis at admission which consisted of six groups: stroke, fracture, lower limb (LL) amputation, LL joint replacement, falls, and others. We included both infarct and hemorrhagic cerebrovascular events under the category ‘stroke’; the majority of limb fractures involved the lower limb; LL amputations included forefoot, below-knee and above knee amputations; LL joint replacements included hip and knee joint replacements; falls included all cases where falls were the primary reason for admission for rehabilitation.

### Statistical Analysis

We used descriptive staftistics to examine differences in socio-demographic characteristics and discharge destination. Fisher’s exact test was performed to test for association between 2×2 categorical variables while the Chi-square test was performed on the other categorical variables. The independent t-test was performed on variables with a normal distribution and the Mann-Whitney U test was performed on skewed variables to test for differences in means and medians respectively across two groups. Analysis of variance (ANOVA) was performed on data with a normal distribution and the Kruskal Wallis test was performed on data with a skewed distribution to test for differences between three or more groups based on their primary diagnosis at admission. In handling outliers, natural log transformation was performed on R-effectiveness and R-efficiency. However, as this could only be performed on positive outcomes, those who had deteriorated in their functional status would be missing. We also performed analyses by shifting all data points to the right by the same factor and took natural log transformation. However, the odds ratio became less interpretable. Outliers were defined as having an absolute value greater than three times the standard deviation from the mean and these were dropped for certain variables (R-effectiveness (n = 1503), R-efficiency (n = 1503), length of stay (n = 1389), and time from onset of principal diagnosis to rehabilitation (n = 1297)). A backward stepwise logistic regression model was used to predict the discharge destinations of home and nursing home. The treating hospital and year of admission were adjusted as clustering effects. The Hosmer-Lemeshow goodness-of-fit chi-square statistic was used to test for goodness-of-fit. [30] The likelihood ratio test was used to test for comparisons across nested models. Akaike information criterion (AIC) and Bayesian information criterion (BIC) were computed in the model summary. We used STATA version 11 (StataCorp LP,USA) for statistical analysis and the significance level was set at P<0.05.

## Results

### Study Population

Among the 17,046 inpatient admissions for rehabilitation, 2,271 had missing information on either admission BI (n = 665) or discharge BI (n = 1,904) scores or both which resulted in missing values in R-effectiveness or R-efficiency. The bulk of missing information was at discharge. Among those with a missing discharge BI score, 823 (43%) patients were discharged back to acute hospitals and only 197 (10%) were discharged to nursing homes; 2,269 (13.3%) second and subsequent admissions were excluded; 1,702 patients were further excluded due to extreme values for R-effectiveness (n = 1503), R-efficiency (n = 1503), length of stay (n = 1389) or time to rehabilitation (n = 1297), leaving 9,518 patients as the final study population who were discharged home (n = 8,594; 90.3%) or to a nursing home (n = 924; 9.7%). Other discharge destinations such as sheltered home (n = 37), other community hospital (n = 31), death in community hospital (n = 12), acute hospital (n = 1,182) and discharge against doctor’s advice (n = 24) were excluded.

### Univariate Analyses

The overall median R-effectiveness and R-efficiency were 31.6% and 13.9 units per month respectively. Compared to those discharged home, patients placed in nursing homes had 33% lower median R-effectiveness (22 vs. 33%), 29% lower median R-efficiency (10 vs. 14), were significantly older (mean age: 77 vs. 73 yrs old), had lower mean admission ADL scores (40 vs. 48), a longer median length of stay (40 vs. 33 days) and a longer time to rehabilitation (19 vs. 15 days) (**Table 1** **),** had a higher proportion without a caregiver (28 vs. 7%), being single (21 vs. 7%), widowed (50 vs. 47%), separated/divorced (5 vs. 3%), with chronic pulmonary disease (6 vs. 4%), dementia (23 vs. 10%), lower proportion with diabetes (29 vs. 38%), hypertension (61 vs. 66%) and hyperlipidemia (25 vs. 30%) (**Table 1** **, Table S1 in File S1**). After adjusting for clustering effects (year of admission and hospital clusters), those admitted for LL arthroplasty had 0.43 (95% CI: 0.24–0.78, p = 0.006) odds of being discharged to a nursing home compared to stroke patients, whereas those admitted due to falls had 2.12 (95% CI: 1.41–3.17, p<0.001) odds of being discharged to a nursing home compared to stroke patients (**Table S2 in File S1**).

**Table 1:** Descriptive table by primary diagnosis at admission to Singapore community hospitals from 1996 to 2005.

Variables	Total (n = 9518)		Stroke (n = 3903)		Fracture (n = 2982)		Lower limb amputation (n = 193)		Lower limb joint arthroplasty (n = 303)		Falls(n = 171)		Others (n = 1966)	
	Home(n = 8594)	NH(n = 924)	Home(n = 3560)	NH(n = 343)	Home(n = 2711)	NH(n = 271)	Home(n = 169)	NH(n = 24)	Home(n = 291)	NH(n = 12)	Home(n = 139)	NH(n = 32)	Home(n = 1724)	NH(n = 242)
Gender, n (%)														
Male	3402 (40)	409 (44)	1746 (49)	170 (50)	680 (25)	80 (30)	90 (53)	11 (46)	45 (15)	3 (25)	56 (40)	13 (41)	785 (46)	132 (55)
Female	5192 (60)	515(56)*	1814 (51)	173 (50)	2031 (75)	191 (70)	79 (47)	13 (54)	246 (85)	9 (75)	83 (60)	19 (59)	939 (54)	110(45)*
Ethnicity, n (%)														
Chinese	7544 (88)	868 (94)	3041 (85)	326 (95)	2472 (91)	254 (94)	144 (85)	23 (96)	262 (90)	10 (83)	124 (89)	31 (97)	1501 (87)	224 (93)
Malay	598 (7)	27 (3)	328 (9)	8 (2)	127 (5)	10 (4)	13 (8)	0 (0)	9 (3)	1 (8)	9 (6)	1 (3)	112 (7)	7 (3)
Indian	352 (4)	18 (2)	157 (4)	7 (2)	82 (3)	4 (1)	11 (7)	1 (4)	10 (3)	0 (0)	3 (2)	0 (0)	89 (5)	6 (2)
Others	100 (1)	11 (1)*	34 (1)	2 (1)*	30 (1)	3 (1)	1 (1)	0 (0)	10 (3)	1 (8)	3 (2)	0 (0)	22 (1)	5 (2)*
Community hospital, n (%)														
A	4775 (56)	493 (53)	1907 (54)	186 (54)	1467 (54)	143 (53)	104 (62)	14 (58)	207 (71)	10 (83)	68 (49)	15 (47)	1022 (59)	125 (52)
B	1794 (21)	248 (27)	853 (24)	94 (27)	466 (17)	52 (19)	45 (27)	8 (33)	46 (16)	2 (17)	46 (33)	16 (50)	338 (20)	76 (31)
C	1795 (21)	125 (14)	773 (22)	44 (13)	643 (24)	51 (19)	18 (11)	2 (8)	38 (13)	0 (0)	15 (11)	0 (0)	308 (18)	28 (12)
D	230 (3)	58 (6)*	27 (1)	19 (6)*	135 (5)	25 (9)*	2 (1)	0 (0)	0 (0)	0 (0)	10 (7)	1 (3)	56 (3)	13 (5)*
Marital status, n (%)														
Married	3782 (44)	220 (24)	1949 (55)	104 (30)	876 (32)	45 (17)	89 (53)	10 (42)	110 (38)	2 (17)	51 (37)	4 (13)	707 (41)	55 (23)
Single	562 (7)	193 (21)	129 (4)	62 (18)	202 (7)	45 (17)	10 (6)	3 (13)	25 (9)	2 (17)	17 (12)	8 (25)	179 (10)	73 (30)
Widowed	4027 (47)	466 (50)	1386 (39)	155 (45)	1570 (58)	173 (64)	61 (36)	10 (42)	147 (51)	8 (67)	68 (49)	20 (63)	795 (46)	100 (41)
Separated/Divorced	223 (3)	45 (5)*	96 (3)	22 (6)*	63 (2)	8 (3)*	9 (5)	1 (4)	9 (3)	0 (0)	3 (2)	0 (0)*	43 (2)	14 (6)*
Caregiver, n (%)														
No	583 (7)	258 (28)	134 (4)	80 (23)	200 (7)	70 (26)	12 (7)	4 (17)	41 (14)	6 (50)	16 (12)	13 (41)	180 (10)	85 (35)
Yes	8011 (93)	666(72)*	3426 (96)	263(77)*	2511 (93)	201(74)*	157 (93)	20 (83)	250 (86)	6 (50)*	123 (88)	19 (59)*	1544 (90)	157(65)*
Religion, n (%)														
No	845 (10)	152 (16)	348 (10)	51 (15)	265 (10)	42 (16)	14 (8)	5 (21)	23 (8)	1 (8)	16 (12)	7 (22)	179 (10)	46 (19)
Yes	7749 (90)	772(84)*	3212 (90)	292(85)*	2446 (90)	229(85)*	155 (92)	19 (79)†	268 (92)	11 (92)	123 (88)	25 (78)	1545 (90)	196(81)*
Government subsidy, n (%)														
Low or no subsidy	4068 (47)	204 (22)	1955 (55)	96 (28)	1165 (43)	50 (18)	74 (44)	9 (38)	151 (52)	3 (25)	37 (27)	3 (9)	686 (40)	43 (18)
High subsidy (C)	4526 (53)	720(78)*	1605 (45)	247(72)*	1546 (57)	221(82)*	95 (56)	15 (63)	140 (48)	9 (75)†	102 (73)	29 (91)*	1038 (60)	199(82)*
Charlson comorbidy (CCMI), n (%)														
0	1730 (20)	155 (17)	20 (1)	1 (0)	1139 (42)	96 (35)	3 (2)	0 (0)	156 (54)	6 (50)	30 (22)	7 (22)	382 (22)	45 (19)
1–3	3961 (46)	458 (50)	1476 (41)	148 (43)	1281 (47)	148 (55)	112 (66)	11 (46)	123 (42)	5 (42)	65 (47)	18 (56)	904 (52)	128 (53)
4–6	2594 (30)	281 (30)	1906 (54)	177 (52)	248 (9)	25 (9)	49 (29)	11 (46)	10 (3)	1 (8)	37 (27)	5 (16)	344 (20)	62 (26)
≥7	309 (4)	30 (3)†	158 (4)	17 (5)	43 (2)	2 (1)†	5 (3)	2 (8)	2 (1)	0 (0)	7 (5)	2 (6)	94 (5)	7 (3)†
Chronic pulmonary disease, n (%)														
No	8260 (96)	870 (94)	3453 (97)	324 (94)	2607 (96)	260 (96)	164 (97)	24 (100)	287 (99)	12 (100)	132 (95)	30 (94)	1617 (94)	220 (91)
Yes	334 (4)	54 (6)*	107 (3)	19 (6)*	104 (4)	11 (4)	5 (3)	0 (0)	4 (1)	0 (0)	7 (5)	2 (6)	107 (6)	22 (9)†
Dementia, n (%)														
No	7773 (90)	712 (77)	3252 (91)	279 (81)	2459 (91)	198 (73)	164 (97)	20 (83)	284 (98)	11 (92)	100 (72)	22 (69)	1514 (88)	182 (75)
Yes	821 (10)	212(23)*	308 (9)	64 (19)*	252 (9)	73 (27)*	5 (3)	4 (17)*	7 (2)	1 (8)	39 (28)	10 (31)	210 (12)	60 (25)*
Peripheral vascular disease, n (%)														
No	8106 (94)	875 (95)	3419 (96)	331 (97)	2603 (96)	266 (98)	76 (45)	10 (42)	281 (97)	12 (100)	128 (92)	30 (94)	1599 (93)	226 (93)
Yes	488 (6)	49 (5)	141 (4)	12 (4)	108 (4)	5 (2)†	93 (55)	14 (58)	10 (3)	0 (0)	11 (8)	2 (6)	125 (7)	16 (7)
Renal Disease, n (%)														
No	8204 (95)	881 (95)	3444 (97)	332 (97)	2622 (97)	264 (97)	154 (91)	23 (96)	282 (97)	11 (92)	135 (97)	28 (88)	1567 (91)	223 (92)
Yes	390 (5)	43 (5)	116 (3)	11 (3)	89 (3)	7 (3)	15 (9)	1 (4)	9 (3)	1 (8)	4 (3)	4 (13)*	157 (9)	19 (8)
Hemiplegia, n (%)														
No	4588 (53)	486 (53)	238 (7)	23 (7)	2418 (89)	230 (85)	155 (92)	12 (50)	279 (96)	10 (83)	101 (73)	22 (69)	1397 (81)	189 (78)
Yes	4006 (47)	438 (47)	3322 (93)	320 (93)	293 (11)	41 (15)*	14 (8)	12 (50)*	12 (4)	2 (17)*	38 (27)	10 (31)	327 (19)	53 (22)*
Diabetes, n (%)														
No	5362 (62)	654 (71)	2005 (56)	231 (67)	1924 (71)	218 (80)	17 (10)	3 (13)	226 (78)	11 (92)	95 (68)	25 (78)	1095 (64)	166 (69)
Yes	3232 (38)	270(29)*	1555 (44)	112(33)*	787 (29)	53 (20)*	152 (90)	21 (88)	65 (22)	1 (8)	44 (32)	7 (22)	629 (36)	76 (31)
Hypertension, n (%)														
No	2952 (34)	365 (40)	746 (21)	86 (25)	1298 (48)	135 (50)	70 (41)	10 (42)	106 (36)	6 (50)	48 (35)	16 (50)	684 (40)	112 (46)
Yes	5642 (66)	559 (61)*	2814 (79)	257 (75)†	1413 (52)	136 (50)	99 (59)	14 (58)	185 (64)	6 (50)	91 (65)	16 (50)	1040 (60)	130 (54)†
Hyperlipidemia, n (%)														
No	6000 (70)	695 (75)	1919 (54)	203 (59)	2282 (84)	238 (88)	119 (70)	17 (71)	228 (78)	11 (92)	114 (82)	27 (84)	1338(78)	199 (82)
Yes	2594 (30)	229 (25)*	1641 (46)	140 (41)†	429 (16)	33 (12)	50 (30)	7 (29)	63 (22)	1 (8)	25 (18)	5 (16)	386 (22)	43 (18)
Age, mean (SD)	73 (11)	77 (10)*	71 (10)	74 (10)*	76 (12)	81 (9)*	67 (11)	69 (9)	71 (10)	76 (8)	79 (10)	80 (8)	74 (12)	77 (11)*
Admission BI, mean (SD)	48 (25)	40 (24)*	41 (26)	32 (23)*	51 (23)	40 (21)*	57 (20)	40 (23)*	68 (18)	60 (16)	53 (21)	52 (24)	53 (25)	47 (24)*
Discharge BI, mean (SD)	64 (28)	53 (27)*	56 (30)	44 (28)*	68 (25)	55 (25)*	69 (23)	54 (28)*	82 (17)	67 (19)*	69 (22)	64 (23)	68 (26)	60 (26)*
Time to rehababilitation, median (IQR)	15 (10–22)	19 (13–26)*	13 (9–20)	18 (12–29)*	16 (12–22)	19 (13–24)*	20 (14–29)	24 (16–31)	12 (9–18)	16 (12–27)†	13 (9–19)	15 (12–22)*	18 (12–26)	20 (13–30)*
Length of stay, median (IQR)	33 (23–46)	40 (29–55)*	36 (25–49)	44 (32–57)*	33 (24–46)	37 (29–53)*	46 (33–60)	47 (25–60)	24 (18–35)	33 (24–48)†	32 (25–44)	45 (33–61)*	30 (20–40)	36 (26–50)*
R-effectiveness, median (IQR)	33 (10–57)	22 (4–42)*	26 (5–51)	15 (1–35)*	38 (15–62)	25 (8–47)*	27 (8–50)	25 (4–53)	48 (28–73)	14 (0–40)*	33 (14–58)	21 (4–39)*	36 (12–61)	26 (7–46)*
R-efficiency, median (IQR)	14 (5–25)	10 (2–19)*	13 (3–24)	8 (0–17)*	16 (7–25)	12 (4–21)*	8 (3–15)	10 (4–18)	18 (11–28)	4 (0–13)*	15 (6–28)	6 (1–18)*	16 (6–27)	10 (4–21)*

* P<0.05;

† 0.05≤P<0.10.

These variables could be group into four broad groups (social, rehabilitation, medical conditions and confounders): social variables were marital status, caregiver availability and government subsidy class; rehabilitation variables were admission BI scores, time to rehabilitation, R-effectiveness and R-efficiency; medical conditions were primary diagnosis at admission, dementia, peripheral vascular disease and hemiplegia; and confounders were age, gender ethnicity and religion.

### Multivariate Analyses

Those who were admitted to a nursing home had a longer hospital length of stay as they were required to wait for their placements. Thus this phenomenon of lengthened stay could be an artifact and thus we excluded it when fitting our best fit regression model. In the multivariate analyses, after adjusting for clustering (year of admission and hospital), every unit increase in functional rehabilitation outcomes R-effectiveness or R-efficiency, the odds of nursing home admission was 0.99 (95% CI: 0.99–1.00, p<0.001) and 0.99 (95% CI: 0.98–1.00, p<0.001) times respectively (**Table 2**). Patients admitted with LL amputation or falls had an odds of nursing home placement of 2.75 (1.59–4.77, p<0.001) and 1.65 (1.02–2.67, p = 0.043) compared to stroke patients respectively. The strongest predictor of discharge to a nursing home observed was the absence of a caregiver. Patients without a caregiver had an odds of nursing home placement of 4.39 (95% CI: 3.51–5.48, p<0.001)) times compared to those with a caregiver. Patients who were widowed or separated/divorced or single had a respective odds of nursing home placement of 4.14 (3.13–5.49, p<0.001), 3.46 (2.32–5.16, p<0.001) and 1.60 (1.30–1.96, p<0.001) compared to those who were currently married. For every 1 year increase in age, the odds of being discharged to a nursing home was 1.03 (95% OR: 1.02–1.04, p<0.001). Patients with dementia or hemiplegia had a respective odds of nursing home placement of 1.85 (1.52–2.25, p<0.001) and 1.38 (1.09–1.74, p = 0,007) compared to those without the disease. Every 1 day increase in time to rehabilitation, the odds of nursing home placement was 1.02 (1.01–1.02, p<0.001). Chinese had the highest odds of being discharged to a nursing home compared to Malays or Indians. Female had lower odds of nursing home placement of 0.71 (95%:0.59–0.84, p<0.001) compared to males (**Table 2**).

**Table 2:** Odd ratios of nursing home placement by primary diagnosis at admission in Singapore community hospitals from 1996 to 2005 (multivariate analyses).

	Total (n = 9518)	Stroke (n = 3903)	Fracture (n = 2982)	Lower limb amputation (n = 193)	Lower limb joint arthroplasty (n = 303)	Falls (n = 171)	Others (n = 1966)
Primary diagnosis at admission							
Stroke	1.00 (ref)						
Fracture	1.19 (0.91–1.55)						
LL Amputation	2.75 (1.59–4.77)*						
LL Arthroplasty	0.85 (0.44–1.65)						
Falls	1.65 (1.02–2.67)*						
Others	1.42 (1.10–1.84)*						
Gender							
Male	1.00 (ref)		1.00 (ref)			1.00 (ref)	1.00 (ref)
Female	0.71 (0.59–0.84)*		0.62 (0.44–0.87)*			0.22 (0.07–0.76)*	0.65 (0.46–0.92)*
Ethnicity							
Chinese	1.00 (ref)	1.00 (ref)					1.00 (ref)
Malay	0.34 (0.22–0.52)*	0.21 (0.10–0.44)*					0.36 (0.16–0.84)*
Indian	0.46 (0.28–0.76)*	0.43 (0.19–0.99)*					0.52 (0.22–1.27)
Others	0.81 (0.40–1.65)	0.23 (0.04–1.3)†					1.58 (0.53–4.68)
Marital status							
Married	1.00 (ref)	1.00 (ref)	1.00 (ref)			1.00 (ref)	1.00 (ref)
Single	4.14 (3.13–5.49)*	6.31 (3.97–10.04)*	3.88 (2.22–6.76)*			0.95 (0.10–9.36)	4.08 (2.46–6.77)*
Widowed	1.60 (1.30–1.96)*	1.45 (1.08–1.93)*	1.63 (1.09–2.46)*			8.67 (1.97–38.2)*	1.39 (0.92–2.12)
Separated/Divorced	3.46 (2.32–5.16)*	3.68 (2.05–6.61)*	2.70 (1.09–6.70)*				5.2 (2.41–11.2)*
Caregiver							
Yes	1.00 (ref)	1.00 (ref)	1.00 (ref)	1.00 (ref)	1.00 (ref)	1.00 (ref)	1.00 (ref)
No	4.39 (3.51–5.48)*	5.25 (3.54–7.76)*	4.72 (3.17–7.01)*	7.26 (1.47–35.97)*	6.23 (1.48–26.16)*	26.6 (3.5–202.2)*	3.44 (2.27–5.21)*
Government Subsidy							
Low or no subsidy	1.00 (ref)	1.00 (ref)	1.00 (ref)				1.00 (ref)
High subsidy (C)	2.55 (2.09–3.12)*	2.29 (1.67–3.14)*	3.24 (2.22–4.75)*				2.42 (1.58–3.72)*
Religion							
No	1.00 (ref)						
Yes	0.81 (0.65–1.01)†						
Chronic pulmonary disease							
No		1.00 (ref)					
Yes		1.84 (1.03–3.28)*					
Dementia							
No	1.00 (ref)	1.00 (ref)	1.00 (ref)				1.00 (ref)
Yes	1.85 (1.52–2.25)*	1.76 (1.25–2.48)*	2.25 (1.59–3.17)*				1.79 (1.22–2.62)*
Hemiplegia							
No	1.00 (ref)		1.00 (ref)	1.00 (ref)			
Yes	1.38 (1.09–1.74)*		1.49 (1–2.22)†	11.1 (3.44–35.8)*			
Peripheral vascular disease							
No	1.00 (ref)		1.00 (ref)				
Yes	0.72 (0.5–1.02)†		0.43 (0.17–1.11)†				
Renal Disease							
No						1.00 (ref)	
Yes						17.1 (1.7–170.4)*	
Age	1.03 (1.02–1.04)*	1.03 (1.01–1.04)*	1.05 (1.03–1.06)*				1.04 (1.02–1.05)*
Admission BI	0.98 (0.98–0.98)*	0.98 (0.97–0.98)*	0.98 (0.97–0.99)*	0.95 (0.92–0.98)*	0.94 (0.90–0.98)*	0.98 (0.96–1.00)†	0.98 (0.98–0.99)*
Time to rehabilitation	1.02 (1.01–1.02)*	1.02 (1.01–1.03)*					1.01 (1.00–1.03)*
R-effectiveness	0.99 (0.99–1.00)*		0.99 (0.98–0.99)*				0.99 (0.98–0.99)*
R-efficiency	0.99 (0.98–1.00)*	0.97 (0.96–0.98)*			0.86 (0.79–0.95)*	0.95 (0.91–0.99)*	

* P<0.05 (logistic regression adjusted for community hospital and year of admission).

† 0.05≤P<0.10 (logistic regression adjusted for community hospital and year of admission).

The best fit model including social variables, rehabilitation variables medical variables and confounders explained 20.1% of variation whereas the best fit model with length of stay explained 19.5%. After adjusting for clustering variables, the largest percentage variation was explained by social variables (9.48%), followed by rehabilitation variables (6.10%) and confounders (4.05%) as well as medical conditions of patients at admission (3.62%) (**Table 3**).

**Table 3:** Percentage variation explained by predictors in the overall model.

	Total population (n = 9,518) (20.1% of variation was explained)	Univariate % variation	Joint % variation
Clustering variables	Year of admission	0.01%	1.10%
	Community Hospital	1.10%	
Social variables	Marital status	4.39%	9.48%
	Caregiver	5.43%	
	Government Subsidy	3.79%	
Rehabilitation variables	Admission Barthel Index Score	1.60%	6.10%
	Time to rehabilitation	1.30%	
	Rehabilitation Effectiveness	1.80%	
	Rehabilitation Efficiency	1.40%	
Confounders	Age	1.71%	4.05%
	Gender	0.12%	
	Ethnicity	0.68%	
	Religion	0.57%	
Medical variables	Primary diagnosis at admission	0.79%	3.62%
	Dementia	2.07%	
	Peripheral vascular disease	0.00%	
	Hemiplegia	0.00%	

Upon stratification by primary diagnosis at admission, the best fit model with R-effectiveness and R-efficiency as predictors was favoured in the stroke (Pseudo R^2^ = 22.3%), fracture (Pseudo R^2^ = 19.5%), joint replacement (Pseudo R^2^ = 33.8%), and others (Pseudo R^2^ = 19.4%) groups, whereas LL amputation (Pseudo R^2^ = 29.5%) and falls (Pseudo R^2^ = 34.8%) favoured length of stay as predictors in the model. The lowest AIC and BIC scores produced consistent models (**Table S3 in File S1**).

Sensitivity analysis was performed assuming all patients who were discharged to acute hospitals were finally discharged home. The odds ratios of social factors, such as caregiver availability and marital status, had a slight reduction in magnitude. Otherwise, results were very similar with our current analyses.

Sensitivity analysis was also performed assuming that all patients who were discharged to acute hospitals were eventually discharged to nursing homes. The odds ratio of social factors, such as caregiver availability and marital status, had a reduction in magnitude but they remained statistically significant. However, REy was no longer statistically significant and peripheral vascular disease became risk conferring. Other significant variables were similar to our current analyses.

## Discussion

The decision of discharge destination of post-rehabilitation patients is complex with the interplay of many variables. These often include age, clinical condition, functional status at admission as well as family support and financial factors. In our study, the odds of nursing home admission was increased in Chinese, males, single or widowed or separated/divorced, those who are highly subsidized for hospital care, dementia, lower ADL scores on admission, lower R-effectiveness or R-efficiency measures on discharge, primary diagnosis groups such as LL amputation and falls compared to stroke and the absence of caregivers.

### Rehabilitation Outcomes and Activities of Daily Living

Patients with poorer admission functional scores were more likely to be discharged to a nursing home as they would be more reliant on caregivers for support in activities of daily living. This finding is well known as several other studies observed that patients who were dependent in three or more activities of daily living had higher odds of admission to nursing homes.[16], [31]–[33] However, little is known about whether a patient’s R-effectiveness and R-efficiency measures predict nursing home admission. Although a longer hospital length of stay is often a strong predictor of nursing home placement, there could be reverse causation as an increased length of stay was likely to be as a result of waiting for their nursing home placement. In addition, for most disease groups, R-effectiveness or R-efficiency were better predictors compared to a patient’s length of stay with higher R^2^ and/or lower AIC and BIC scores. Patients who had higher R-effectiveness or R-efficiency measures were less likely to be discharged to a nursing home, even after adjusting for functional status at admission: every 20 units increased in R-effectiveness or R-efficiency reduced the odds of nursing home admission by 18.2%.

### Primary Diagnosis at Admission

As most studies on nursing home admissions were conducted for specific disease groups such as stroke, fracture and arthroplasty patients, it is uncertain which groups were more likely to be admitted to a nursing home as the comparison of odds between different disease groups was not previously possible. For our model, we used stroke as a reference group as it was the most prevalent condition in our study. In our overall model, we found that patients admitted to community hospitals with a primary diagnosis of LL amputation or falls had odds of nursing home placement of 2.75 (95%CI: 1.59–4.77) and 1.65 (95%: 1.02–2.67) compared to stroke patients respectively. A sensitivity analysis was performed by further stratifying the primary diagnosis into their subcategories (i.e. stroke: infarct, haemorrhage or both, fracture: femoral or vertebral, amputation: below or above knee). Compared to patients with cerebral infarction as the reference group, the odds ratios of femoral and vertebral fractures were not statistically different, however below and above knee amputations had increased odds of nursing home admission by 191% and 378% respectively. Falls and other diseases had statistically higher odds of nursing home admission by 67% and 45% respectively when compared to patients admitted due to cerebral infarction.

### Caregiver Availability and Marital Status

Previous studies have consistently shown that social support factors such as older married adults with more living children had lower odds of nursing home admission, whereas those who lived alone had twice the odds of nursing home admission.[16], [34]–[36]. Married older persons have approximately half the risk of nursing home admission as unmarried people (e.g., widowed, never married, divorced, and separated). [9], [10] The probability of institutionalisation increases with age. [11] A population-based survey of 1,079 elderly aged ≥60 in Singapore on the prevalence of late-life functional disability found that the overall prevalence of functional disability increased with age and was particularly more dramatic for those aged ≥80 [12], [13].

In our study, one of the strongest factors for nursing home placement was caregiver unavailability (OR = 4.39). Even after adjusting for caregiver unavailability, single and separated/divorced persons had an odds of nursing home placement of 4.14 (95%CI: 3.13–5.49) and 3.46 (95%CI: 2.32–5.16) respectively when compared to married persons. With the projected population ageing and the changing family structure having more singles, divorces and smaller family size, [37] the demand for nursing home admission is expected to rise dramatically in Singapore. As such, in a recent budget speech by the Ministerial Committee on Aging in Singapore, a plan was announced to increase the number of nursing home beds by 70% from 9,000 in 2012 to 15,600 by 2020 [38].

### Cognitive Function

In a US study, cognitive impairment was found to be a strong predictor of nursing home placements with as many as 90% of dementia patients institutionalized before death. [39] Systematic reviews have also shown that having both dementia and cognitive impairment predisposes patients for institutionalization in the elderly. [16], [35] A recent meta-analysis predicting nursing home admission in the US [16] reported that patients who had cognitive impairment had a significantly higher likelihood of nursing home admission (OR = 2.54). Van Baalan et al also showed that patients with poorer cognitive status at time of discharge had a higher likelihood of being admitted to an institution. [40] In our study, dementia was a significant predictor for nursing home admission in the overall population (OR = 1.85, 95%CI: 1.52–2.25) with the highest odds found in patients admitted for fracture (OR = 2.25, 95%CI: 1.59–3.17), followed by other diseases (OR = 1.79, 95%CI: 1.22–2.62) and stroke (OR = 1.76, 95%CI: 1.25–2.48).

### Minority Ethnic Group

Patients of the minority ethnic groups (Malays and Indians) were more likely to be discharged home compared to the Chinese majority, even after adjusting for caregiver availability and other confounders. This may be due to residual confounding of a larger family size that was not fully adjusted for in the caregiver variables. Malays and Indians tend to have more children [37], live with their extended families and may have “stronger family ties” which make them more reluctant and less likely to send family members to nursing homes. Family support networks may be stronger and better established in the minority ethnic groups which could explain their increase in the likelihood of being discharged home compared to the Chinese. Our findings are similar to a US study by Graham et al [41] that found that ethnic minority groups had a relative advantage compared to non-Hispanic whites as they were more likely to be discharged home. Bhandari et al [42] reported a 70% higher odds of home discharge for non-Hispanic black patients compared to non-Hispanic white patients. This could be due to the possibility that family and social support networks are better established in these groups compared to non-Hispanic whites [43, 44] and minority groups such as Blacks and Hispanics tended to view nursing homes negatively which could also explain the lower nursing home uptakes. [45] Non-Hispanic whites were also more likely to be living alone and responsible for providing their own care, whereas Hispanics were more likely to have care provided by family members or other unpaid persons [46].

### Socio-economic Factors

Patients admitted to high subsidy wards had increased odds of being discharged to nursing homes. Although after adjusting for confounders such as caregiver availability where those in high subsidy wards could afford a maid, the subsidy factor remained significant. Our findings are similar to a previous study by Foley et al. which showed that lower income was a predictor of nursing home admission. [32] In addition, elders from higher income families with the ability to afford paid help or home-care services could avoid nursing home admission but for less privileged elders, it might be more affordable in the long-run to send them to a subsidised nursing home than to use community-based services [13].

### Strength and Limitations

The strengths of this study are the multiple comparisons across different diagnostic groups and exploring R-effectiveness and R-efficiency as predictors of nursing home placement. A limitation is the loss of power of the study upon stratification and comparison between different diagnostic groups. In addition, subjects who were admitted to nursing homes after acute hospital discharge were not considered in our study as follow-up of discharged patients was not done by all community hospitals.

## Conclusions

Predictors of nursing home admission in Singapore were old age, males, Chinese, absence of caregiver, being single/widowed/separated or divorced (compared to married), receiving high subsidies for hospital admission, having dementia, hemiplegia, lower admission BI scores (more dependent at admission), longer time to rehabilitation, and poorer R-effectiveness and R-efficiency. Upon further adjustment for primary diagnosis at admission, patients admitted due to LL amputation or falls had significantly higher odds of being discharged to a nursing home compared to stroke patients, whereas patients admitted due to LL arthroplasty had the lowest odds. With populations around the world ageing rapidly, it is expected that there will be a huge increase in demand for nursing homes. Social factors remained the most important predictor of nursing home placement with the highest odds ratio observed in caregiver availability and marital status and social factors accounts for about 50% of the explained variation in nursing home placement. This is followed by rehabilitation outcomes as better rehabilitation effectiveness and efficiency were associated with decreased odds of nursing home admission. In addition, care planning as well as improving community support can be strengthened to mitigate the demand for nursing homes.

## Supporting Information

File S1 **Supplementary tables (Table S1, Table S2 and Table S3).** Table S1: Descriptive table for diseases by primary diagnosis at admission to Singapore community hospitals from 1996 to 2005. Table S2: Odd ratios of nursing home placement by primary diagnosis at admission in Singapore community hospitals from 1996 to 2005 (univariate analysis). Table S3: Model summary
(DOCX)
