# Supplementary material for: Factors Associated with Nursing Home Placement of All Patients Admitted for Inpatient Rehabilitation in Singapore Community Hospitals from 1996 to 2005: A Disease Stratified Analysis
Source: PLoS One. 2013 Dec 23;8(12):e82697. doi: 10.1371/journal.pone.0082697 (PMC3871833; doi:10.1371/journal.pone.0082697)
Supplement: File S1 — Supplementary tables (Table S1, Table S2 and Table S3). Table S1: Descriptive table for diseases by primary diagnosis at admission to Singapore community hospitals from 1996 to 2005. Table S2: Odd ratios of nursing home placement by primary diagnosis at admission in Singapore community hospitals from 1996 to 2005 (univariate analysis). Table S3: Model summary (DOCX) [file pone.0082697.s001.docx]

| **Table S1.** Descriptive table for diseases by primary diagnosis at admission to Singapore community hospitals from 1996 to 2005. | | | | | | | | | | | | | | |
| --- | --- | --- | --- | --- | --- | --- | --- | --- | --- | --- | --- | --- | --- | --- |
| **Variables** | **Total (n=9518)** | | **Stroke (n=3903)** | | **Fracture (n=2982)** | | **Lower limb amputation (n=193)** | | **Lower limb joint arthroplasty (n=303)** | | **Falls**  **(n=171)** | | **Others (n=1966)** | |
|  | **Home**  **(n=8594)** | **NH**  **(n=924)** | **Home**  **(n=3560)** | **NH**  **(n=343)** | **Home**  **(n=2711)** | **NH**  **(n=271)** | **Home**  **(n=169)** | **NH**  **(n=24)** | **Home**  **(n=291)** | **NH**  **(n=12)** | **Home**  **(n=139)** | **NH**  **(n=32)** | **Home**  **(n=1724)** | **NH**  **(n=242)** |
| AIDS, n(%) |  |  |  |  |  |  |  |  |  |  |  |  |  |  |
| No | 8589(100) | 923(100) | 3557(100) | 342(100) | 2711(100) | 271(100) | 169(100) | 24 (100) | 291 (100) | 12 (100) | 139 (100) | 32 (100) | 1722(100) | 242(100) |
| Yes | 5 (0) | 1 (0) | 3 (0) | 1 (0) | 0 (0) | 0 (0) | 0 (0) | 0 (0) | 0 (0) | 0 (0) | 0 (0) | 0 (0) | 2 (0) | 0 (0) |
| Cerebrovascular disease, n (%) |  |  |  |  |  |  |  |  |  |  |  |  |  |  |
| No | 4331 (50) | 464 (50) | 112 (3) | 14 (4) | 2347 (87) | 223 (82) | 153 (91) | 13 (54) | 277 (95) | 10 (83) | 93 (67) | 22 (69) | 1349 (78) | 182 (75) |
| Yes | 4263 (50) | 460 (50) | 3448 (97) | 329 (96) | 364 (13) | 48 (18) † | 16 (9) | 11 (46)***** | 14 (5) | 2 (17) | 46 (33) | 10 (31) | 375 (22) | 60 (25) |
| Congestive heart failure, n (%) |  |  |  |  |  |  |  |  |  |  |  |  |  |  |
| No | 8137 (95) | 869 (94) | 3427 (96) | 326 (95) | 2588 (95) | 259 (96) | 151 (89) | 22 (92) | 280 (96) | 11 (92) | 128 (92) | 29 (91) | 1563 (91) | 222 (92) |
| Yes | 457 (5) | 55 (6) | 133 (4) | 17 (5) | 123 (5) | 12 (4) | 18 (11) | 2 (8) | 11 (4) | 1 (8) | 11 (8) | 3 (9) | 161 (9) | 20 (8) |
| Connective tissue disease, n (%) |  |  |  |  |  |  |  |  |  |  |  |  |  |  |
| No | 8454 (98) | 906 (98) | 3534 (99) | 337 (98) | 2668 (98) | 265 (98) | 169(100) | 24 (100) | 278 (96) | 12 (100) | 135 (97) | 30 (94) | 1670 (97) | 238 (98) |
| Yes | 140 (2) | 18 (2) | 26 (1) | 6 (2) * | 43 (2) | 6 (2) | 0 (0) | 0 (0) | 13 (4) | 0 (0) | 4 (3) | 2 (6) | 54 (3) | 4 (2) |
| Leukemia, n (%) |  |  |  |  |  |  |  |  |  |  |  |  |  |  |
| No | 8566(100) | 921(100) | 3550(100) | 342(100) | 2702(100) | 271(100) | 168 (99) | 24 (100) | 291 (100) | 12 (100) | 137 (99) | 32 (100) | 1718(100) | 240 (99) |
| Yes | 28 (0) | 3 (0) | 10 (0) | 1 (0) | 9 (0) | 0 (0) | 1 (1) | 0 (0) | 0 (0) | 0 (0) | 2 (1) | 0 (0) | 6 (0) | 2 (1) |
| Lymphoma, n (%) |  |  |  |  |  |  |  |  |  |  |  |  |  |  |
| No | 8566(100) | 920(100) | 3551(100) | 342(100) | 2703(100) | 271(100) | 168 (99) | 24 (100) | 291 (100) | 12 (100) | 137 (99) | 31 (97) | 1716(100) | 240 (99) |
| Yes | 28 (0) | 4 (0) | 9 (0) | 1 (0) | 8 (0) | 0 (0) | 1 (1) | 0 (0) | 0 (0) | 0 (0) | 2 (1) | 1 (3) | 8 (0) | 2 (1) |
| Myocardial infarct, n (%) |  |  |  |  |  |  |  |  |  |  |  |  |  |  |
| No | 8331 (97) | 904 (98) | 3452 (97) | 337 (98) | 2656 (98) | 268 (99) | 157 (93) | 22 (92) | 289 (99) | 12 (100) | 136 (98) | 32 (100) | 1641 (95) | 233 (96) |
| Yes | 263 (3) | 20 (2) | 108 (3) | 6 (2) | 55 (2) | 3 (1) | 12 (7) | 2 (8) | 2 (1) | 0 (0) | 3 (2) | 0 (0) | 83 (5) | 9 (4) |
| Ulcer disease, n (%) |  |  |  |  |  |  |  |  |  |  |  |  |  |  |
| No | 7582 (88) | 805 (87) | 3175 (89) | 309 (90) | 2446 (90) | 246 (91) | 150 (89) | 22 (92) | 258 (89) | 9 (75) | 116 (83) | 27 (84) | 1437 (83) | 192 (79) |
| Yes | 1012 (12) | 119 (13) | 385 (11) | 34 (10) | 265 (10) | 25 (9) | 19 (11) | 2 (8) | 33 (11) | 3 (25) | 23 (17) | 5 (16) | 287 (17) | 50 (21) |
| Liver disease, n (%) |  |  |  |  |  |  |  |  |  |  |  |  |  |  |
| No | 8520 (99) | 916 (99) | 3539 (99) | 339 (99) | 2698(100) | 270(100) | 167 (99) | 24 (100) | 291 (100) | 12 (100) | 136 (98) | 32 (100) | 1689 (98) | 239 (99) |
| Yes | 74 (1) | 8 (1) | 21 (1) | 4 (1) | 13 (0) | 1 (0) | 2 (1) | 0 (0) | 0 (0) | 0 (0) | 3 (2) | 0 (0) | 35 (2) | 3 (1) |
|  |  |  |  |  |  |  |  |  |  |  |  |  |  |  |
| Malignant tumor, n (%) |  |  |  |  |  |  |  |  |  |  |  |  |  |  |
| No | 8045 (94) | 869 (94) | 3446 (97) | 329 (96) | 2567 (95) | 259 (96) | 160 (95) | 23 (96) | 280 (96) | 12 (100) | 132 (95) | 30 (94) | 1460 (85) | 216 (89) |
| Yes | 549 (6) | 55 (6) | 114 (3) | 14 (4) | 144 (5) | 12 (4) | 9 (5) | 1 (4) | 11 (4) | 0 (0) | 7 (5) | 2 (6) | 264 (15) | 26 (11)† |
| Ischemic heart disease (including myocardial infarct), n(%)^†^ |  |  |  |  |  |  |  |  |  |  |  |  |  |  |
| No | 6510 (76) | 718 (78) | 2684 (75) | 265 (77) | 2156 (80) | 225 (83) | 111 (66) | 13 (54) | 238 (82) | 8 (67) | 98 (71) | 23 (72) | 1223 (71) | 184 (76) |
| Yes | 2084 (24) | 206 (22) | 876 (25) | 78 (23) | 555 (20) | 46 (17) | 58 (34) | 11 (46) | 53 (18) | 4 (33) | 41 (30) | 9 (28) | 501 (29) | 58 (24) |

* P<0.05; †0.05<P<0.10.

^†^not included in CCMI calculation

| **Table S2.** Odd ratios of nursing home placement by primary diagnosis at admission in Singapore community hospitals from 1996 to 2005 (univariate analysis) | | | | | | | |
| --- | --- | --- | --- | --- | --- | --- | --- |
|  | **Total (n=9518)** | **Stroke (n=3903)** | **Fracture (n=2982)** | **Lower limb amputation (n=193)** | **Lower limb joint arthroplasty (n=303)** | **Falls**  **(n=171)** | **Others (n=1966)** |
| Primary diagnosis at admission |  | n.a. | | | | | |
| Stroke | 1.00 (ref) |  |  |  |  |  |  |
| Fracture | 1.02 (0.86-1.21) |  |  |  |  |  |  |
| LL Amputation | 1.42 (0.91-2.21) |  |  |  |  |  |  |
| LL Arthroplasty | 0.43 (0.24-0.78)* |  |  |  |  |  |  |
| Falls | 2.12 (1.41-3.17)* |  |  |  |  |  |  |
| Others | 1.42 (1.19-1.70)* |  |  |  |  |  |  |
| Gender |  |  |  |  |  |  |  |
| Male | 1.00 (ref) | 1.00 (ref) | 1.00 (ref) | 1.00 (ref) | 1.00 (ref) | 1.00 (ref) | 1.00 (ref) |
| Female | 0.84 (0.73-0.96)* | 0.99 (0.79-1.24) | 0.82 (0.62-1.08) | 1.3 (0.55-3.08) | 0.58 (0.15-2.27) | 0.89 (0.40-2.00) | 0.71 (0.54-0.93)* |
| Ethnicity |  |  |  |  |  |  |  |
| Chinese | 1.00 (ref) | 1.00 (ref) | 1.00 (ref) | 1.00 (ref) | 1.00 (ref) | 1.00 (ref) | 1.00 (ref) |
| Malay | 0.39 (0.26-0.58)* | 0.22 (0.11-0.46)* | 0.77 (0.40-1.49) | nc | 3.95 (0.42-36.88) | 0.46 (0.05-3.9) | 0.40 (0.18-0.87)* |
| Indian | 0.44 (0.27-0.71)* | 0.43 (0.20-0.92)* | 0.47 (0.17-1.28) | 0.64 (0.08-5.25) | nc | nc | 0.46 (0.20-1.06)† |
| Others | 0.99 (0.53-1.86) | 0.53 (0.12-2.28) | 0.96 (0.29-3.20) | nc | 3.02 (0.31-29.06) | nc | 1.80 (0.67-4.85) |
| Marital status |  |  |  |  |  |  |  |
| Married | 1.00 (ref) | 1.00 (ref) | 1.00 (ref) | 1.00 (ref) | 1.00 (ref) | 1.00 (ref) | 1.00 (ref) |
| Single | 5.58 (4.50-6.91)* | 8.38 (5.81-12.10)* | 4.14 (2.66-6.45)* | 2.72 (0.63-11.64) | 4.45 (0.57-34.74) | 7.61 (1.89-30.66)* | 5.02 (3.40-7.43)* |
| Widowed | 2.00 (1.69-2.36)* | 2.1 (1.62-2.72)* | 2.19 (1.56-3.08)* | 1.49 (0.58-3.84) | 2.34 (0.48-11.55) | 3.88 (1.22-12.31)* | 1.60 (1.13-2.26)* |
| Separated / Divorced | 3.39 (2.39-4.81)* | 3.97 (2.37-6.66)* | 2.52 (1.14-5.58)* | 1.06 (0.12-9.54) | nc | nc | 3.93 (2.01-7.68)* |
| Caregiver |  |  |  |  |  |  |  |
| Yes | 1.00 (ref) | 1.00 (ref) | 1.00 (ref) | 1.00 (ref) | 1.00 (ref) | 1.00 (ref) | 1.00 (ref) |
| No | 5.13 (4.33-6.07)* | 7.27 (5.33-9.93)* | 4.37 (3.20-5.97)* | 2.53 (0.74-8.74) | 4.60 (1.37-15.38)* | 6.77 (2.56-17.94)* | 4.51 (3.31-6.15)* |
| Religion |  |  |  |  |  |  |  |
| No | 1.00 (ref) | 1.00 (ref) | 1.00 (ref) | 1.00 (ref) | 1.00 (ref) | 1.00 (ref) | 1.00 (ref) |
| Yes | 0.58 (0.48-0.70)* | 0.63 (0.46-0.88)* | 0.62 (0.43-0.88)* | 0.36 (0.12-1.14)† | 1.10 (0.13-9.17) | 0.51 (0.18-1.43) | 0.53 (0.37-0.76)* |
| Government Subsidy |  |  |  |  |  |  |  |
| Low or no subsidy | 1.00 (ref) | 1.00 (ref) | 1.00 (ref) | 1.00 (ref) | 1.00 (ref) | 1.00 (ref) | 1.00 (ref) |
| High subsidy (C) | 3.46 (2.88-4.15)* | 3.26 (2.44-4.36)* | 3.96 (2.79-5.62)* | 1.19 (0.41-3.41) | 3.70 (0.94-14.53)† | 2.08 (0.53-8.14) | 3.23 (2.20-4.75)* |
| Charlson comorbidy (CCMI) |  |  |  |  |  |  |  |
| 0 | 1.00 (ref) | 1.00 (ref) | 1.00 (ref) | empty | 1.00 (ref) | 1.00 (ref) | 1.00 (ref) |
| 1-3 | 1.3 (1.07-1.57)* | 2.62 (0.33-20.48) | 1.37 (1.04-1.79)* | 0.27 (0.04-1.64) | 1.00 (0.29-3.41) | 1.26 (0.46-3.42) | 1.17 (0.82-1.69) |
| 4-6 | 1.21 (0.98-1.48)† | 2.36 (0.3-18.40) | 1.19 (0.75-1.90) | 0.61 (0.1-3.74) | 1.72 (0.18-16.85) | 0.56 (0.16-2.01) | 1.42 (0.93-2.15) |
| >7 | 1.00 (0.67-1.52) | 2.54 (0.31-21.01) | 0.53 (0.13-2.23) | 1.00 (ref) | nc | 0.83 (0.13-5.14) | 0.55 (0.24-1.26) |
| Chronic pulmonary disease |  |  |  |  |  |  |  |
| No | 1.00 (ref) | 1.00 (ref) | 1.00 (ref) | 1.00 (ref) | 1.00 (ref) | 1.00 (ref) | 1.00 (ref) |
| Yes | 1.44 (1.07-1.94)* | 1.87 (1.13-3.10)* | 1.04 (0.55-1.97) | nc | nc | 1.35 (0.26-7.15) | 1.35 (0.83-2.20) |
| AIDS |  |  |  |  |  |  |  |
| No | 1.00 (ref) | 1.00 (ref) | 1.00 (ref) | 1.00 (ref) | 1.00 (ref) | 1.00 (ref) | 1.00 (ref) |
| Yes | 1.46 (0.17-12.54) | 3.26 (0.33-31.69) | nc | nc | nc | nc | nc |
| Congestive heart failure |  |  |  |  |  |  |  |
| No | 1.00 (ref) | 1.00 (ref) | 1.00 (ref) | 1.00 (ref) | 1.00 (ref) | 1.00 (ref) | 1.00 (ref) |
| Yes | 1.11 (0.83-1.49) | 1.19 (0.7-2.03) | 0.98 (0.53-1.81) | 0.73 (0.16-3.39) | 1.4 (0.15-12.79) | 1.36 (0.34-5.43) | 0.86 (0.53-1.4) |
| Cerebrovascular disease |  |  |  |  |  |  |  |
| No | 1.00 (ref) | 1.00 (ref) | 1.00 (ref) | 1.00 (ref) | 1.00 (ref) | 1.00 (ref) | 1.00 (ref) |
| Yes | 1.03 (0.9-1.19) | 0.79 (0.45-1.41) | 1.41 (1.01-1.97)* | 8.29 (3.11-22.09)* | 4.45 (0.82-24.22) † | 1.06 (0.46-2.49) | 1.12 (0.82-1.54) |
| Connective tissue disease |  |  |  |  |  |  |  |
| No | 1.00 (ref) | 1.00 (ref) | 1.00 (ref) | 1.00 (ref) | 1.00 (ref) | 1.00 (ref) | 1.00 (ref) |
| Yes | 1.15 (0.7-1.89) | 1.88 (0.75-4.74) | 1.4 (0.59-3.33) | nc | nc | 2.02 (0.34-11.93) | 0.52 (0.19-1.46) |
| Dementia |  |  |  |  |  |  |  |
| No | 1.00 (ref) | 1.00 (ref) | 1.00 (ref) | 1.00 (ref) | 1.00 (ref) | 1.00 (ref) | 1.00 (ref) |
| Yes | 2.73 (2.30-3.24)* | 2.29 (1.69-3.09)* | 3.62 (2.67-4.90)* | 6.25 (1.53-25.57)* | 3.59 (0.37-35.22) | 1.17 (0.49-2.78) | 2.20 (1.58-3.07)* |
| Hemiplegia |  |  |  |  |  |  |  |
| No | 1.00 (ref) | 1.00 (ref) | 1.00 (ref) | 1.00 (ref) | 1.00 (ref) | 1.00 (ref) | 1.00 (ref) |
| Yes | 1.04 (0.91-1.2) | 1.03 (0.63-1.68) | 1.48 (1.04-2.12)* | 11.5 (4.22-31.3)* | 4.45 (0.82-24.22) † | 1.05 (0.45-2.48) | 1.09 (0.78-1.52) |
| Leukemia |  |  |  |  |  |  |  |
| No | 1.00 (ref) | 1.00 (ref) | 1.00 (ref) | 1.00 (ref) | 1.00 (ref) | 1.00 (ref) | 1.00 (ref) |
| Yes | 0.8 (0.24-2.65) | 0.86 (0.11-6.81) | nc | nc | nc | nc | 1.8 (0.35-9.18) |
| Lymphoma |  |  |  |  |  |  |  |
| No | 1.00 (ref) | 1.00 (ref) | 1.00 (ref) | 1.00 (ref) | 1.00 (ref) | 1.00 (ref) | 1.00 (ref) |
| Yes | 1.02 (0.35-2.93) | 0.92 (0.12-7.38) | nc | nc | nc | 1.45 (0.12-17.26) | 1.32 (0.27-6.37) |
| Myocardial infarct |  |  |  |  |  |  |  |
| No | 1.00 (ref) | 1.00 (ref) | 1.00 (ref) | 1.00 (ref) | 1.00 (ref) | 1.00 (ref) | 1.00 (ref) |
| Yes | 0.68 (0.43-1.08) | 0.55 (0.24-1.26) | 0.55 (0.17-1.76) | 1.11 (0.23-5.36) | nc | nc | 0.73 (0.36-1.49) |
| Peripheral vascular disease |  |  |  |  |  |  |  |
| No | 1.00 (ref) | 1.00 (ref) | 1.00 (ref) | 1.00 (ref) | 1.00 (ref) | 1.00 (ref) | 1.00 (ref) |
| Yes | 0.91 (0.67-1.23) | 0.85 (0.46-1.55) | 0.45 (0.18-1.11) † | 1.09 (0.43-2.75) | nc | 0.71 (0.14-3.52) | 0.9 (0.52-1.55) |
| Ulcer disease |  |  |  |  |  |  |  |
| No | 1.00 (ref) | 1.00 (ref) | 1.00 (ref) | 1.00 (ref) | 1.00 (ref) | 1.00 (ref) | 1.00 (ref) |
| Yes | 1.07 (0.87-1.31) | 0.89 (0.61-1.3) | 0.92 (0.59-1.42) | 0.68 (0.15-3.14) | 2.57 (0.62-10.59) | 0.76 (0.25-2.28) | 1.25 (0.89-1.76) |
| Diabetes |  |  |  |  |  |  |  |
| No | 1.00 (ref) | 1.00 (ref) | 1.00 (ref) | 1.00 (ref) | 1.00 (ref) | 1.00 (ref) | 1.00 (ref) |
| Yes | 0.68 (0.59-0.79)* | 0.63 (0.5-0.8)* | 0.59 (0.43-0.81)* | 0.75 (0.20-2.82) | 0.31 (0.04-2.49) | 0.55 (0.22-1.41) | 0.8 (0.60-1.08) |
| Liver disease |  |  |  |  |  |  |  |
| No | 1.00 (ref) | 1.00 (ref) | 1.00 (ref) | 1.00 (ref) | 1.00 (ref) | 1.00 (ref) | 1.00 (ref) |
| Yes | 0.95 (0.45-1.98) | 1.82 (0.62-5.35) | 0.8 (0.1-6.14) | nc | nc | nc | 0.59 (0.18-1.93) |
| Renal Disease |  |  |  |  |  |  |  |
| No | 1.00 (ref) | 1.00 (ref) | 1.00 (ref) | 1.00 (ref) | 1.00 (ref) | 1.00 (ref) | 1.00 (ref) |
| Yes | 0.99 (0.72-1.38) | 0.89 (0.47-1.68) | 0.75 (0.34-1.65) | 0.45 (0.06-3.62) | 4.63 (0.48-44.57) | 7.25 (1.43-36.82)* | 0.87 (0.53-1.44) |
| Malignant tumor |  |  |  |  |  |  |  |
| No | 1.00 (ref) | 1.00 (ref) | 1.00 (ref) | 1.00 (ref) | 1.00 (ref) | 1.00 (ref) | 1.00 (ref) |
| Yes | 0.91 (0.69-1.22) | 1.26 (0.71-2.23) | 0.82 (0.45-1.5) | 0.87 (0.1-7.43) | nc | 1.12 (0.21-6.02) | 0.69 (0.45-1.06) † |
| Hypertension |  |  |  |  |  |  |  |
| No | 1.00 (ref) | 1.00 (ref) | 1.00 (ref) | 1.00 (ref) | 1.00 (ref) | 1.00 (ref) | 1.00 (ref) |
| Yes | 0.80 (0.70-0.92)* | 0.79 (0.61-1.03) † | 0.92 (0.72-1.19) | 0.91 (0.38-2.22) | 0.69 (0.21-2.27) | 0.51 (0.23-1.13) † | 0.79 (0.6-1.05) |
| Hyperlipidemia |  |  |  |  |  |  |  |
| No | 1.00 (ref) | 1.00 (ref) | 1.00 (ref) | 1.00 (ref) | 1.00 (ref) | 1.00 (ref) | 1.00 (ref) |
| Yes | 0.73 (0.62-0.86)* | 0.71 (0.56-0.89)* | 0.7 (0.48-1.04) † | 0.89 (0.33-2.4) | 0.47 (0.06-3.85) | 0.88 (0.3-2.64) | 0.77 (0.54-1.1) |
| Ischemic heart disease (including myocardial infarct) |  |  |  |  |  |  |  |
| No | 1.00 (ref) | 1.00 (ref) | 1.00 (ref) | 1.00 (ref) | 1.00 (ref) | 1.00 (ref) | 1.00 (ref) |
| Yes | 0.9 (0.76-1.06) | 0.89 (0.68-1.16) | 0.79 (0.57-1.1) | 1.57 (0.64-3.83) | 2.24 (0.63-7.96) | 1 (0.42-2.4) | 0.78 (0.57-1.06) |
| Age | 1.04 (1.03-1.04)* | 1.03 (1.02-1.04)* | 1.05 (1.04-1.07)* | 1.02 (0.98-1.07) | 1.05 (0.98-1.12) | 1.03 (0.98-1.07) | 1.03 (1.02-1.04)* |
| Admission BI | 0.98 (0.98-0.98)* | 0.98 (0.98-0.99)* | 0.97 (0.97-0.98)* | 0.96 (0.94-0.98)* | 0.97 (0.94-1.00) † | 0.99 (0.98-1.01) | 0.99 (0.98-0.99)* |
| Discharge BI | 0.98 (0.98-0.98)* | 0.98 (0.98-0.98)* | 0.98 (0.97-0.98)* | 0.97 (0.95-0.99)* | 0.95 (0.92-0.98)* | 0.99 (0.97-1.00) | 0.99 (0.98-0.99)* |
| Time to rehabilitation | 1.03 (1.02-1.03)* | 1.03 (1.02-1.04)* | 1.02 (1.01-1.03)* | 1.02 (0.98-1.06) | 1.06 (1.01-1.11)* | 1.04 (1.00-1.08) † | 1.01 (1.00-1.02)* |
| Length of stay | 1.02 (1.02-1.03)* | 1.03 (1.02-1.03)* | 1.02 (1.01-1.03)* | 0.99 (0.96-1.01) | 1.03 (0.99-1.07) | 1.04 (1.02-1.07)* | 1.02 (1.01-1.03)* |
| R-effectiveness | 0.98 (0.98-0.99)* | 0.98 (0.98-0.99)* | 0.98 (0.98-0.99)* | 0.99 (0.98-1.01) | 0.97 (0.94-0.99)* | 0.98 (0.97-1.00)* | 0.99 (0.98-0.99)* |
| R-efficiency | 0.97 (0.97-0.98)* | 0.96 (0.96-0.97)* | 0.98 (0.97-0.99)* | 1.01 (0.97-1.05) | 0.89 (0.82-0.96)* | 0.96 (0.92-0.99)* | 0.98 (0.97-0.99)* |

* **P<0.05** (logistic regression adjusted for community hospital and year of admission); odds ratios reported.

†0.05<P<0.10 (logistic regression adjusted for community hospital and year of admission); odds ratios reported.

n.a: non-applicable, nc: non-calculable due to small sample size.

| **Table S3.** Model summary | | | | | | | |
| --- | --- | --- | --- | --- | --- | --- | --- |
|  | **Total (n=9518)** | **Stroke (n=3903)** | **Fracture (n=2982)** | **Lower limb amputation (n=193)** | **Lower limb joint arthroplasty (n=303)** | **Falls**  **(n=171)** | **Others (n=1966)** |
| **R-effectiveness + Clustering effects:** |  |  |  |  |  |  |  |
| Goodness of fit p-value | 0.100 | 0.542 | 0.077 | 0.722 | 0.740 | 1.000 | 0.931 |
| Log-likelihood | -2921.9 | -1102.2 | -878.8 | -71.6 | -42.4 | -75.2 | -707.5 |
| Pseudo-R^2^ | 0.037 | 0.051 | 0.032 | 0.009 | 0.131 | 0.050 | 0.035 |
| AIC | 5855.7 | 2216.4 | 1769.6 | 153.1 | 92.9 | 160.5 | 1427.0 |
| BIC | 5898.7 | 2254.0 | 1805.6 | 169.4 | 107.2 | 175.7 | 1460.5 |
| **R-efficiency + Clustering effects:** |  |  |  |  |  |  |  |
| Goodness of fit p-value | 0.285 | 0.445 | 0.954 | 0.710 | 0.534 | 0.943 | 0.001 |
| Log-likelihood | -2945.4 | -1104.5 | -893.3 | -71.9 | -40.4 | -73.6 | -711.2 |
| Pseudo-R^2^ | 0.029 | 0.049 | 0.017 | 0.005 | 0.174 | 0.070 | 0.030 |
| AIC | 5902.7 | 2220.9 | 1798.5 | 153.7 | 88.7 | 157.3 | 1434.5 |
| BIC | 5945.7 | 2258.5 | 1834.6 | 170.0 | 103.0 | 172.5 | 1468.0 |
| **R-effectiveness + R-efficiency + Clustering effects:** |  |  |  |  |  |  |  |
| Goodness of fit p-value | 0.647 | 0.216 | 0.042 | 0.817 | 0.244 | 0.943 | 0.789 |
| Log-likelihood | -2919.3 | -1099.1 | -878.6 | -70.9 | -40.3 | -73.6 | -706.7 |
| Pseudo-R^2^ | 0.037 | 0.054 | 0.033 | 0.018 | 0.176 | 0.070 | 0.036 |
| AIC | 5852.6 | 2212.2 | 1771.3 | 153.8 | 90.5 | 159.3 | 1427.3 |
| BIC | 5902.8 | 2256.0 | 1813.3 | 173.4 | 108.4 | 177.6 | 1466.4 |
| **Best fit model (including either R-effectiveness or R-efficiency):** |  |  |  |  |  |  |  |
| Goodness of fit p-value | n.a | 0.150 | 0.002 | 0.177 | 0.903 | 0.840 | 0.138 |
| Log-likelihood | Best fit | -903.1 | -731.1 | -52.5 | -33.7 | -52.0 | -592.1 |
| Pseudo-R^2^ | Model | 0.223 | 0.195 | 0.273 | 0.309 | 0.303 | 0.193 |
| AIC | Includes | 1844.2 | 1496.2 | 119.0 | 79.5 | 127.0 | 1222.1 |
| BIC | Both | 1963.4 | 1598.2 | 141.7 | 101.0 | 159.8 | 1328.2 |
| **Best fit model (including both R-effectiveness and R-efficiency):** |  |  |  |  |  |  |  |
| Goodness of fit p-value | 0.260 | 0.129 | 0.003 | 0.764 | 0.873 | 0.800 | 0.343 |
| Log-likelihood | -2422.3 | -902.1 | -731.0 | -52.3 | -32.3 | -54.7 | -591.2 |
| Pseudo-R^2^ | 0.201 | 0.223 | 0.195 | 0.275 | 0.338 | 0.303 | 0.194 |
| AIC | 4900.5 | 1844.2 | 1498.0 | 122.7 | 80.7 | 133.3 | 1222.5 |
| BIC | 5101.0 | 1969.6 | 1606.0 | 151.9 | 109.3 | 169.7 | 1334.1 |
| **Best fit model (LOS: substituting R-effectiveness + R-efficiency):** |  |  |  |  |  |  |  |
| Goodness of fit p-value | 0.067 | 0.949 | 0.074 | 0.232 | 0.481 | 0.719 | 0.427 |
| Log-likelihood | -2442.3 | -910.2 | -737.0 | -48.8 | -39.4 | -49.2 | -595.0 |
| Pseudo-R2 | 0.195 | 0.216 | 0.189 | 0.295 | 0.161 | 0.348 | 0.188 |
| AIC | 4938.7 | 1860.4 | 1508.1 | 111.7 | 88.9 | 122.4 | 1227.9 |
| BIC | 5132.0 | 1985.8 | 1610.1 | 133.6 | 106.0 | 157.8 | 1334.0 |
